# Potential missed opportunities for diagnosis of cryptococcosis and the association with mortality: A cohort study

**DOI:** 10.1016/j.eclinm.2020.100563

**Published:** 2020-10-07

**Authors:** Ana S. Salazar, Matthew R. Keller, Margaret A. Olsen, Katelin B. Nickel, Ige A. George, Lindsey Larson, William G. Powderly, Andrej Spec

**Affiliations:** a Division of Infectious Diseases, Department of Medicine, Washington University School of Medicine in St. Louis, 4523 Clayton Ave., Campus Box 8051, St Louis, MO 63110-0193, United Statess; b Division of Public Health Sciences, Department of Surgery, Washington University School of Medicine in St. Louis, 660 S. Euclid Ave, Campus Box 8100, St. Louis, MO 63110-0193, USA

**Keywords:** Cryptococcus, Delayed diagnosis, Misdiagnosis, Administrative data

## Abstract

**Background:**

Cryptococcosis is one of the most common life-threatening opportunistic mycoses worldwide. Insidious presentation and slow onset of symptoms make it difficult to recognize, complicating the diagnostic process. Delays in diagnosis may lead to increased mortality. We aim to determine the frequency of missed opportunities for diagnosis of cryptococcosis and its effects on mortality.

**Methods:**

To estimate the proportion of individuals with a potentially missed diagnosis for cryptococcosis in hospitalized patients, we conducted a retrospective cohort study using the Healthcare Cost and Utilization Project State Inpatient Databases from 2005 to 2015 from eight states. All hospitalized adult patients diagnosed with cryptococcal infection or cryptococcal meningitis were included. Potentially missed diagnoses were defined as admissions coded for a procedure or diagnosis suggestive of cryptococcosis in the 90-days prior to the initial cryptococcosis admission. Generalized estimating equations models were used to evaluate the association between underlying comorbidities and potential missed diagnosis of cryptococcosis and 90-day all-cause in-hospital mortality.

**Findings:**

Of 5,354 patients with cryptococcosis, 2,445 (45·7%) were people living with HIV (PLWH). Among PLWH, 493/2,445 (20·2%) had a potentially missed diagnosis, of which 83/493 (16·8%) died while hospitalized compared with 265/1,952 (13·6%) of those without a potentially missed diagnosis (relative risk [RR] 1·04, 95% CI 0·99–1·09). Among HIV-negative patients, 977/2,909 (33·6%) had a potentially missed diagnosis, of which 236/977 (24·2%) died while hospitalized compared with 298/1,932 (15·4%) of those not missed (RR 1·12, 95% CI 1·07–1·16).

**Interpretation:**

Missed opportunities to diagnose cryptococcosis are common despite highly efficacious diagnostic tests and are associated with increased risk of 90-day mortality in HIV-negative patients. A high index of clinical suspicion is paramount to promptly diagnose, treat, and improve cryptococcosis-related mortality.

**Funding:**

National Center for Advancing Translational Sciences, Washington University Institute of Clinical and Translational Sciences, and the Agency for Healthcare Research and Quality.

**Research in context**

**Evidence before this study**

Cryptococcosis is one of the most common life-threatening opportunistic mycoses worldwide.

Diagnosis is readily made using the cryptococcal antigen test, which is inexpensive, rapid, and highly sensitive.

Early diagnosis during the asymptomatic phase of illness in cryptococcosis has been associated with lower mortality.

**Added value of this study**

To our knowledge, this is the first and largest epidemiological study to report the proportion of potentially missed opportunities for cryptococcosis diagnosis and its association with mortality.

The overall missed Cryptococcal diagnosis rate was 27%. Potential missed opportunities for diagnosis were associated with an absolute 8.8% increased risk of 90-day mortality in the non-HIV cohort.

Metastatic cancer, lymphoma, and neurological disorders were associated with increased risk of possible missed diagnosis in both people living with HIV and HIV-negative patients.

**Implications of all the available evidence**

Missed opportunities to diagnose cryptococcosis in the hospital setting are common, particularly among HIV-negative patients.

Missed opportunities are associated with significantly increased risk of 90-day mortality in the HIV-negative group.

We highlight the need for a higher index of suspicion for cryptococcosis, and emphasize the importance of early cryptococcal antigen testing to accelerate diagnosis and decrease cryptococcal-related mortality.

Alt-text: Unlabelled box

## 1 Introduction

Cryptococcosis is one of the most common life-threatening opportunistic mycoses worldwide. Variable presentation and slow onset of symptoms make it difficult to recognize, especially in populations with lesser known risk factors, resulting in high morbidity and mortality [1,2].

Several studies have shown poorer outcomes for cryptococcosis in the general population when compared to people living with HIV (PLWH) or patients with a solid organ transplant [3], [4], [5], [6]. The root causes of poor outcomes remain unclear, but a delay in diagnosis of cryptococcosis has been suggested as a possible reason for worse outcomes seen in HIV-negative patients [6,7]. Delayed diagnosis is a major concern, because dissemination and progression to more severe disease may occur, resulting in higher risk for mortality [6,7]. Diagnosis of cryptococcosis using cryptococcal antigen (CrAg) testing is inexpensive, easy to use, and highly reliable [8], [9], [10], suggesting that delayed diagnosis is more often due to lack of consideration of cryptococcosis in the differential diagnosis rather than availability of an affordable test.

We used population-based administrative billing data from multiple US states to investigate the association between missed diagnosis and mortality risk. Administrative billing data are a part of the information stored in patient electronic health records. Billing data include the information generated for health insurers for billing purposes containing a summary of inpatient care transformed into medical codes, using a standardized language of ICD9 or ICD10 [11]. These data were used to estimate the proportion of PLWH and HIV-negative individuals with a potentially missed opportunity for diagnosis of cryptococcosis and identify relevant patient characteristics and underlying conditions associated with increased risk of delayed diagnosis.

## 2 Methods

### 2.1 Data source and patient population

We used the Healthcare Cost and Utilization Project (HCUP) [11] State Inpatient Databases (SID) from Arkansas (2008–2015), California (2005–2011), Florida (2005–2015), Massachusetts (2010–2015), Maryland (2013–2015), Nebraska (2005–2015), New York (2005–2015), and Wisconsin (2013–2015). The SID are census-level uniformly formated databases available through the Agency for Healthcare Research and Quality (AHRQ). They consist of all-payer inpatient hospital discharge data within a year from all community acute-care hospitals within a participating state. The SID contain information on patient demographics, diagnoses and procedures, payment sources, and discharge status.

The selection of states and years was limited to those available for purchase with the availability of a patient-level encrypted identifier, which is needed to follow hospitalizations over time for a single individual. The states included represent 30% of the total US population and a wide variety of geographic regions and cryptococcosis epidemiology. We excluded patients diagnosed with cryptococcosis within the first three months of the first year from each state, as well as new diagnoses in the last three months of the last year available from each state. This was to ensure a minimum of 90 days of retrospective analysis and 90 days of follow-up.

Eligibility criteria for the study were: 1) a discharge International Classification of Diseases, 9th edition, Clinical Modification (ICD-9-CM) diagnosis code for cryptococcal infection (117·5) or cryptococcal meningitis (321·0); 2) age ≥ 18 years; 3) residence in the state of hospitalization; 4) availability of linkage with patient-level encrypted identifier; 5) non-missing gender and death status. New York and Nebraska do not provide a patient-level encrypted identifier for PLWH due to privacy concerns. Therefore, only data for HIV-negative patients were included from New York and Nebraska and no analyses for PLWH could be performed. If the patient had multiple hospitalizations coded for cryptococcosis, the earliest coded encounter was identified as the index hospitalization. Due to the limited nature of the dataset, and as the analysis of the data denotes minimal risk to the patient, this study was considered exempt from ethics board review by the Washington University School of Medicine Human Research Protection Office.

### 2.2 Definitions

The index hospitalization was defined as the admission during which the diagnosis of cryptococcosis was coded. We defined a potential missed diagnosis of cryptococcosis as a hospitalization that met the following criteria: 1) the patient was hospitalized in the 90-days prior to the admission date and was discharged to home, or was transferred into the index hospitalization after a stay of seven or more days without coding for cryptococcosis, and 2) the patient had one or more procedures or diagnoses related to a central nervous system (CNS), respiratory condition, or a diagnosis of non-specific signs of infection suggestive of cryptococcosis (Supplementary Table 1). Hospitalizations of more than seven days that resulted in transfer to the index hospitalization were categorized as a potential missed diagnosis of cryptococcosis because the physicians had sufficient time to diagnose cryptococcosis, as the median time to diagnosis of cryptococcosis is five days from admission [12].

To determine which CNS, respiratory, and non-specific signs of infection conditions were suggestive of cryptococcosis, three physicians, who are experts in the management of cryptococcosis, independently reviewed the ICD-9-CM procedure and diagnosis codes coded during all hospitalizations in the 90 days prior to the cryptococcosis admission and classified only those highly suggestive of cryptococcosis into the three groups (CNS, respiratory, or non-specific signs/symptoms of cryptococcosis). A select group of codes were chosen, resulting in a more restrictive group of codes suggestive of cryptococcosis. Each physician expert reviewed this code list three times, each time eliminating, but never adding, codes to provide a conservative estimate of a missed diagnosis of cryptococcosis. The patients were classified as missed using hierarchical categories, with cryptococcosis-related procedure and diagnosis codes for CNS disease being preferred to those of respiratory conditions, which in turn were preferred to non-specific signs of infection (Supplementary Table 1 and Supplementary Table 2).

Comorbidities were identified within 90 days prior to and during the index hospitalization. Variables were selected based on those classically associated with cryptococcus, including leukemia, cellular immunodeficiency, history of solid organ transplant, and neurologic and autoimmune disorders (Supplementary Table 3) [13,14]. We also supplemented those with commonly available comorbidities from the well validated algorithm of Elixhauser, as they have been shown to be good predictors of in-hospital mortality [15,16]. The neurologic and autoimmune disorders variables were a modification of two Elixhauser variables, rheumatoid arthritis and other neurological disorders, respectively (Supplementary Table 3).

### 2.3 Statistical analysis

After calculating the proportion of patients with potential missed diagnosis for cryptococcosis, we performed univariate and multivariable analyses to evaluate the association between underlying conditions and (1) a potential missed diagnosis of cryptococcosis; and (2) 90-day all-cause in-hospital mortality. All analyses were performed separately for PLWH and non-HIV patients due to significant underlying differences [3,12,17]. Chi-square and Fisher's exact test were used for univariate analyses, as appropriate. Predictive variables with *p* <0·2 in univariate analysis or with clinical/biological plausibility were assessed in multivariable generalized estimating equations models, accounting for clustering at the hospital-level [18]. Backwards variable elimination was used to develop the most parsimonious models, retaining only variables with *p* < 0·05 in the final models. For the mortality models, missed opportunity was included in the model regardless of significance in order to assess its impact; comorbidities were to adjust for potential confounding. In the HIV-negative mortality analysis, the impact of age on mortality was not linear. To adjust for this, splines were used to model age, but were not necessary in the model of PLWH, where age was not associated with mortality. Results from all regression models are presented as relative risk (RR) and 95 % confidence intervals (CI).

Kaplan-Meier plots were used to visualize the time from index admission date (hospitalization in which diagnosis of cryptococcosis was first coded) to death by the presence of missed opportunity to diagnose cryptococcosis, stratified by HIV status. The model was adjusted for potential confounders. We verified the assumption of proportional hazards for the missed using visual inspection. All analyses were performed using SAS v·9·4 statistical software (SAS Institute Inc., Cary, NC).

## 3 Role of the funding source

No funding source had any role in the design or conduct of the study, collection, analysis and interpretation of the data, or writing of the manuscript. All investigators had full access to the data and had final responsibility for the decision to submit the manuscript for publication.

## 4 Results

### 4.1 Cohort demographics

During the study period, 5,354 patients were coded for cryptococcosis during an inpatient admission. Cases were distributed across the following states: California (42·9%), Florida (31·9%), New York (12·6%), Arkansas (4·8%), Massachusetts (2·8%), Maryland (2·8%), Nebraska (1·1%), and Wisconsin (1·1%). The baseline characteristics of the cryptococcosis populations are shown in Table 1. Less than half of the cohort were PLWH (45·7%), of which a large majority (78·5%) were between ages 18–50 years. PLWH were most commonly African American (44·0%), residing in the lowest income quartile (51·4%) and had Medicaid listed as the primary payer (54·0%). More than 70% of HIV-negative patients were over the age of 50. The HIV-negative group was predominantly white (52·5%), with equal distribution across the income quartiles, and 46·9% had Medicare listed as the primary payer.

**Table 1:** Baseline Characteristics of 5354 Patients Diagnosed with Cryptococcosis Stratified by HIV Status.

Characteristic	PLWH N (%)	HIV-Negative N (%)
Total	2,445 (100%)	2,909 (100%)
Age (years)		
18–40	960 (39·3)	381 (13·1)
41–50	958 (39·2)	461 (15·9)
51–60	411 (16·8)	677 (23·3)
60–70	94 (3·8)	668 (23·0)
	22 (0·9)	722 (24·8)
Sex		
Female	431 (17·6)	1,104 (38·0)
Race		
White	629 (25·7)	1,527 (52·5)
African American	1,076 (44·0)	430 (14·8)
Hispanic	618 (25·3)	543 (18·7)
Other	122 (5·0)	409 (14·1)
Insurance		
Medicaid	1,083 (44·3)	505 (17·4)
Medicare	459 (18·8)	1,363 (46·9)
Self-Pay/Private/Others	903 (36·9)	1,041 (35·8)
Median Household Income State Quartile		
0–25th	1,256 (51·4)	815 (28·0)
26–50th	530 (21·7)	752 (25·9)
51–75th	406 (16·6)	702 (24·1)
76–100th	201 (8·2)	562 (19·3)
Missing	52 (2·1)	78 (2·7)
Number of Patients by State		
Arkansas	72 (2·9)	184 (6·3)
California	1,284 (52·5)	1,013 (34·8)
Florida	931 (38·1)	779 (26·8)
Massachusetts	45 (1·8)	103 (3·5)
Maryland	100 (4·1)	51 (1·8)
Nebraska	··	57 (2·0)
New York	··	677 (23·3)
Wisconsin	13 (0·5)	45 (1·6)
Selected Comorbidities		
Neurological Disorders*	124 (5·1)	187 (6·4)
Chronic Pulmonary Disease	66 (2·7)	224 (7·7)
Diabetes Mellitus	228 (9·3)	1,054 (36·2)
Hypertension	670 (27·4)	1,746 (60·0)
Autoimmune Disorders*	51 (2·1)	304 (10·4)
Lymphoma	53 (2·2)	229 (7·9)

⁎ Modified from Elixhauser – Neurological disorders excludes the following conditions: altered mental status, aphasia, convulsions, cerebral degeneration; Autoimmune disorders include: psoriatic arthritis, psoriasis, reactive arthritis, Kawasaki, Takayasu, granulomatosis w/polyangiitis, eosinophilic (Wegener) giant cell arteritis, hypersensitivity angiitis, Cerebral arthritis, arteritis not otherwise specified, Sjögren, Inflammatory bowel disease, lupus, sarcoid, Cushing disease. Abbreviations: HIV, human immunodeficiency viruses; PLWH, people living with HIV.

### 4.2 Identification of potential missed opportunities for diagnosis of cryptococcosis

**Fig. 1:**
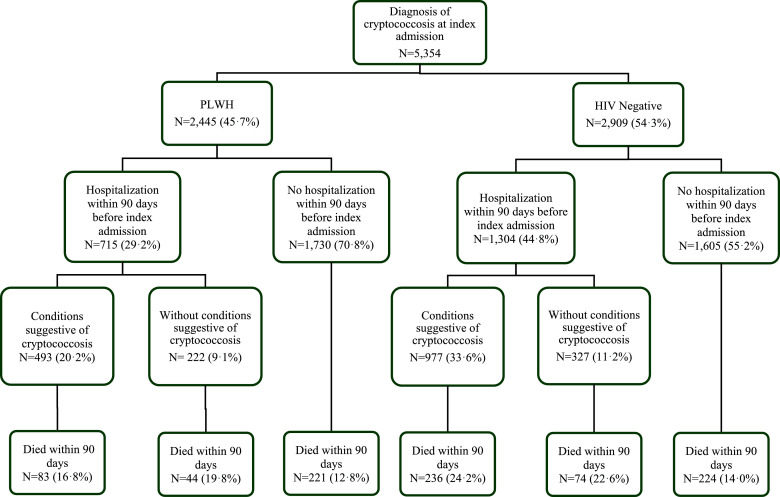
Flowchart for characterization of patients with cryptococcal disease stratified by HIV status. Potentially missed diagnoses for cryptococcal disease (CD) are common in both Persons Living with HIV (PLWH) and in HIV-negative populations. In the HIV-negative population, a potentially missed CD diagnosis is associated with higher mortality.

A total of 1,470/5,354 (27·5%) patients had a potentially missed diagnosis of cryptococcosis (Fig. 1). Of the patients considered to have a potentially missed cryptococcosis diagnosis, 778/1,470 (52·9%) were coded for a CNS-related condition(s), 575/1,470 (39·1%) were coded for pulmonary-related conditions, and 117/1,470 (8·0%) were coded for non-specific signs or symptoms of infection (Supplementary Table 2). The most common CNS-related conditions coded for individuals with a potential missed diagnosis of cryptococcosis included lumbar puncture (214/1,47014·6%), encephalopathy (235/1,470, 16·0%), and focal neurological diseases (140/1,470, 9·5%). The most common pulmonary-related conditions during prior hospitalization were pneumonia without a specified organism (490/1,470, 33·3%) and mediastinal and pleural disease (85/1,470, 5·8%).

Among PLWH, 715/2,445 (29·2%) had a prior hospitalization within 90 days, and 493/2,445 (20·2%) were coded for a condition during a prior hospitalization considered to represent a potentially missed diagnosis of cryptococcosis. Among HIV-negative patients, 1,304/2,909 (44·8%) had a prior hospitalization within 90 days, of which 977/2,909 (33·6%) had a potential missed diagnosis of cryptococcosis (Fig. 1). For the subgroup of patients that were initially treated at another hospital, and then transferred to the index hospital in which the diagnosis of cryptococcosis was established (*n* = 88), the median length of stay was 14 days (IQR 10, 19).

### 4.3 Risk of missed diagnosis of cryptococcosis during a prior hospitalization

Univariate predictors of a potential missed diagnosis of cryptococcosis in PLWH and HIV-negative patients are listed in Supplementary Table 4. In multivariable analysis in the PLWH population, several factors were independently associated with potentially missed diagnosis of cryptococcosis, including metastatic cancer, lymphoma, other neurological disorders, chronic pulmonary disease, nutritional deficiency anemia, drug abuse (excluding alcohol), and congestive heart failure (Table 2). In HIV-negative patients, factors associated with potentially missed diagnosis of cryptococcosis in multivariable analysis included metastatic cancer, lymphoma, deficiency anemia, leukemia, chronic liver disease, weight loss, other neurological disorders, and chronic pulmonary disease (Table 2).

**Table 2:** Multivariable Analysis of Risk for Missed Diagnosis within 90 Days Prior to the Cryptococcosis Admission by HIV Status.

	PLWH		HIV-NEGATIVE	
	RR (95% CI)	P	RR (95% CI)	P
History of Solid Organ Transplant			1·22 (1·06–1·41)	0·004
Congestive Heart Failure	1·55 (1·20–2·02)	0·001	1·21 (1·09–1·35)	<0·001
Chronic Liver Disease	1·35 (1·14–1·60)	<0·001	1·44 (1·26–1·65)	<0·001
Hypertension	1·41 (1·21–1·63)	<0·001	1·30 (1·15–1·47)	<0·001
Metastatic Cancer	2·53 (1·73–3·71)	<0·001	1·57 (1·33–1·85)	<0·001
Other Neurological Disorders*	1·73 (1·41–2·13)	<0·001	1·41 (1·22–1·64)	<0·001
Autoimmune Disorders†			1·39 (1·23–1·59)	<0·001
Pulmonary Circulation Disorders			1·31 (1·15–1·50)	<0·001
Chronic Pulmonary Disease	1·66 (1·43–1·91)	<0·001	1·40 (1·26–1·55)	<0·001
Peripheral Vascular Disease			1·24 (1·07–1·43)	0·004
Diabetes Mellitus			1·15 (1·05–1·27)	0·004
Hypothyroidism	1·57 (1·05–2·33)	0·027	1·20 (1·07–1·34)	0·002
Leukemia			1·48 (1·21–1·80)	<0·001
Lymphoma	1·94 (1·40–2·69)	<0·001	1·54 (1·33–1·78)	<0·001
Obesity	1·46 (1·06–2·03)	0·022	1·24 (1·09–1·41)	0·001
Deficiency Anemia	1·59 (1·35–1·86)	<0·001	1·52 (1·36–1·70)	<0·001
Drug Abuse	1·56 (1·32–1·84)	<0·001	1·27 (1·05–1·54)	0·014
Alcohol Abuse	1·29 (1·05–1·59)	0·015		
Weight Loss			1·43 (1·30–1·58)	<0·001

⁎ Other neurological disorders include: altered mental status, aphasia, convulsions, cerebral degeneration.

† Autoimmune disorders include: psoriatic arthritis, psoriasis, reactive arthritis, Kawasaki, Takayasu, granulomatosis w/polyangiitis, eosinophilic (Wegener) giant cell arteritis, hypersensitivity angitis, Cerebral arthritis, arteritis not otherwise specified, Sjögren, Inflammatory bowel disease, lupus, sarcoid, and Cushing disease. Abbreviations: HIV, human immunodeficiency viruses; PLWH, people living with HIV; RR, relative risk; CI, confidence intervals.

### 4.4 Risk of 90-day in-hospital mortality

The 90-day mortality rate in PLWH was 14·2% (348/2,445) (Fig. 1). We found a trend of increased all-cause in-hospital mortality in patients with a potentially missed diagnosis of cryptococcosis compared to patients without a missed diagnosis (16·8% vs. 13·6%, respectively; RR 1·04, 95% CI 0·99–1·09).

The 90-day mortality rate in HIV-negative patients was 18·4% (534/2,909). Mortality was significantly higher in patients with a potentially missed diagnosis of cryptococcosis compared to those without a missed diagnosis of cryptococcosis (24·2% vs. 15·4%% RR 1·12; 95% CI, 1·07–1·16). Patients with a prior hospitalization in the past 90 days that was not coded for a potentially related diagnosis of cryptococcosis or procedure also had significantly higher 90-day all-cause mortality 22= compared to those with no prior hospitalization (22·6% vs. 14·0%, respectively; *p* < 0·001).

**Fig. 2:**
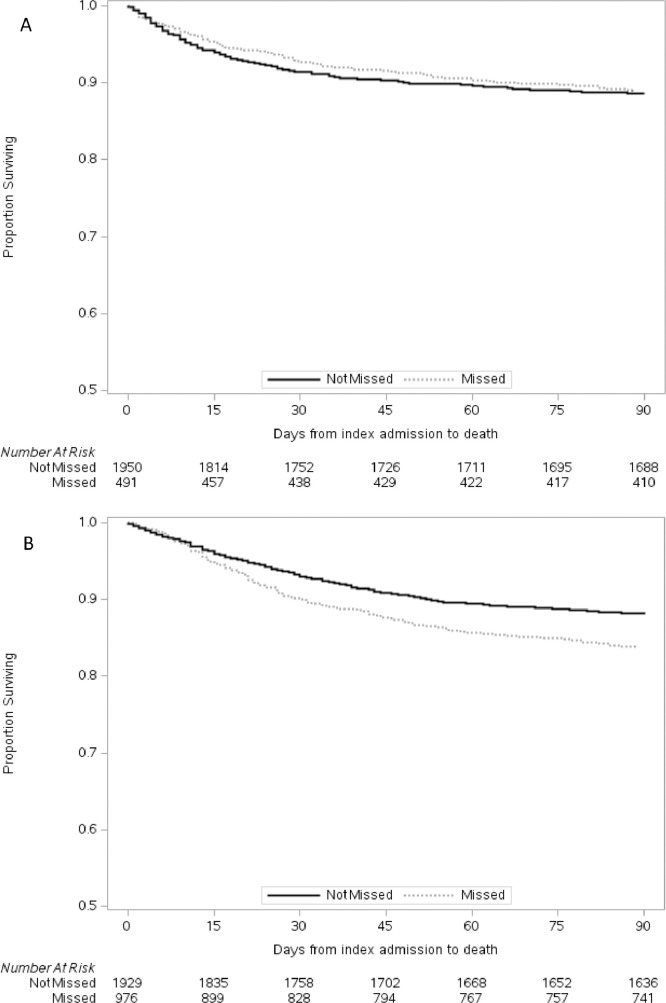
Cox proportional hazards curves comparing time from index admission to death by potential missed diagnosis of cryptococcal disease for (a) persons living with HIV and (b) HIV-negative patients. Patients with a potential missed opportunity for diagnosis of Cryptococcus have a higher mortality amongst those that are HIV-negative, but not those that are HIV positive.

Univariate predictors of 90-day mortality in PLWH and HIV-negative patients are listed in Supplementary Table 5. In the PLWH population, potentially missed diagnosis of cryptococcosis was associated with marginally increased risk of 90-day mortality compared to patients without a potentially missed diagnosis of cryptococcosis (log-rank test *p* = 0·075) (Fig. 2A). In contrast, potentially missed diagnosis of cryptococcosis was associated with significantly increased risk of 90-day mortality in the HIV-negative population compared to patients without a potentially missed diagnosis of cryptococcosis (log rank *p*<0·001) (Fig. 2B).

In multivariable analysis among PLWH, factors independently associated with significantly increased risk of 90-day mortality included rheumatoid arthritis, peripheral vascular disease, metastatic cancer, congestive heart failure, other neurological disorders, chronic liver disease, and hypertension (Table 3). Potentially missed diagnosis of cryptococcosis was not associated with increased risk of mortality in PLWH after adjusting for other factors (RR, 0·97; 95% CI, 0·75–1·25). In the HIV-negative cohort, significantly increased risk of 90-day mortality was associated with chronic liver disease, weight loss, congestive heart failure, and potentially missed diagnosis of cryptococcosis (RR, 1·27; 95% CI, 1·09–1·49). A history of solid organ transplant was associated with significantly lower risk of mortality in the HIV-negative population (RR, 0·59; 95% CI, 0·44–0·80) (Table 3).

**Table 3:** Multivariable analysis of risk for mortality within 90 days after the cryptococcosis admission by HIV status.

	PLWH		HIV-NEGATIVE*	
	RR (95% CI)	P	RR (95% CI)	P
Potential Missed Opportunity for Diagnosis of Cryptococcosis †	0·97 (0·75–1·25)	0·807	1·27 (1·09–1·49)	0·003
History of Solid Organ Transplant			0·59 (0·44–0·80)	<0·001
Congestive Heart Failure	2·12 (1·54–2·91)	<0·001	1·31 (1·11–1·5)	0·002
Chronic Liver Disease	1·42 (1·10–1·83)	0·008	2.11 (1·77–2·52)	<0·001
Weight Loss			1.41 (1·21–1·67)	<0·001
Hypertension	1·37 (1·13–1·66)	0·001		
Metastatic Cancer	2·32 (1·21–4·45)	0·011		
Rheumatoid Arthritis or Collagen Vascular Diseases‡	2·73 (1·02–7·33)	0·047		
Peripheral Vascular Disease	2·68 (1·74–4·15)	<0·001		
Other Neurological Diseases§	1·71 (1·22–2·39)	0·002		

⁎ HIV-negative model was adjusted for age (splines used,7 knots), age was not a significant variable in the model of PLWH.

† Variable was forced into the model as it was the primary exposure.

‡ Modified from Elixhauser - domain excludes Systemic Lupus Erythematous.

§ Other neurological disorders include: altered mental status, aphasia, convulsions, cerebral degeneration Abbreviations: HIV, human immunodeficiency viruses; PLWH, people living with HIV; RR, relative risk; CI, confidence intervals.

## 5 Discussion

To our knowledge, this is the first epidemiological study reporting the proportion of potentially missed opportunities for diagnosis of cryptococcosis and its association with mortality in HIV-negative patients. In our cohort, the 90-day in-hospital mortality rate (16·5%) was similar to mortality rates reported in previous studies (16%−31%) [3,6,12,17,[19], [20], [21]]. Not surprisingly, HIV-negative patients had higher mortality than PLWH (18·4% vs 14·2%, respectively). Previous studies have shown similar associations of mortality due to cryptococcosis by HIV status in the US (22–35% vs. 15–19%, respectively) [3,19,21] and in Taiwan (30% vs. 5%, respectively) [5]. Our study only allowed us to assess inpatient mortality, thus accounting for the lower observed mortality, comparable with mortality seen in other studies using SID [20]. Missed opportunities for diagnosis in HIV-negative patients was associated with a 9% increase of absolute risk of mortality [8], [9], [10]. This is consistent with the absolute difference in mortality previously observed in the literature between the groups of PLWH and those that are HIV-negative (8%−20%), suggesting that this difference may exist due to delays in diagnosis [3,17,19].

Other potential factors contributing to poorer outcomes in this group may include the number and severity of underlying comorbidities. Comparable with previous studies, our results showed that patients with organ failure syndromes, such as congestive heart failure and chronic liver disease, had increased 90-day mortality [6,19,20]. It is also possible that PLWH experience better outcomes because the immune function of PLWH is more readily reversible, with proper intervention, when compared to other immunocompromising conditions. However, this explanation is less likely because the majority of cryptococcosis mortality occurs soon after diagnosis, and PLWH with cryptococcosis appear to have high mortality after the initial phase of disease [21].

Infectious diseases with sub-acute and pleomorphic clinical presentations pose a challenge for timely diagnosis, especially in populations with lower incidence. We found that missed opportunities for diagnosis of cryptococcosis were common in the hospital setting, with 27·5% of all cases of cryptococcosis having a potential missed opportunity for diagnosis during a prior hospitalization(s), and the HIV-negative group having a higher rate of missed diagnosis than PLWH (33·6% vs 20·2%, respectively). Miller et al. found a comparable missed diagnosis rate of 25% for tuberculosis [22] utilizing HCUP data from both inpatient and emergency department encounters [23]. Similarly, in two studies examining missed opportunities in coccidioidomycosis, diagnosis was delayed by more than a month in 46% and 43% of patients [24,25]. In smaller cohorts of patients with cryptococcal infection, missed opportunities for diagnosis have been shown to be associated with delayed initiation of treatment, ultimately leading to further dissemination and progression of disease, permanent neurological deficits, and higher all-cause [3] and disease-specific mortality [3,4]. Conversely, early diagnosis during the asymptomatic phase of illness is associated with lower mortality [26,27], and has formed the basis for cryptococcal antigen (CrAg) screening of HIV-positive patients at highest risk of cryptococcosis at time of presentation (PLWH and a CD4 count less than 100 cells/mL) [28] which has been associated with a 28% survival benefit [29].

Time to diagnosis can be significantly longer among HIV-negative patients compared to PLWH and has been proposed as a cause of increased mortality. [3,12,30] Brizendine et al. reported time from initial presentation to diagnosis was longer in HIV-negative patients compared to PLWH (26–68 vs 22 days), even though HIV-negative patients are generally symptomatic for a longer duration prior to diagnosis (44 days vs 19 days) [6]. These observations have been repeated in other studies [12,19,31], and the less “classical” underlying conditions such as cirrhosis, immunosuppressant medications and even those who are immunocompetent are associated with more delays in diagnosis [19,[32], [33], [34]]. As the majority of the global burden of cryptococcosis is in PLWH [2,20,35], clinicians have a higher level of suspicion when considering cryptococcosis in HIV in PLWH. As such, PLWH are diagnosed earlier and have lower mortality [3,19,21]. Conversely, patients with cirrhosis, for example, are at an increased risk for cryptococcosis [35], however, most physicians do not consider them to be an at-risk population, resulting in delayed diagnoses and mortality as high as 80% [12].

We investigated the association between the type of insurance coverage and risk of missed diagnosis or mortality. We hypothesized that private insurance served as a surrogate marker for higher socioeconomic status. For this purpose, patients were categorized into two groups: those who were uninsured or had government provided insurance (i.e. Medicaid), and those with private insurance. Previous studies have found that PLWH with cryptococcosis and with no insurance or the government option had higher mortality rates compared to those with private insurance [21]. In our cohort, having Medicaid or being uninsured was associated with an increased risk of missed diagnosis in PLWH in the univariate analysis. However, no effect was seen after adjustment in the multivariate analysis.

With the advent of effective antiretroviral therapy, the risk of cryptococcosis in PLWH has decreased significantly but has remained constant in HIV-negative patients [20]. As a result, non-HIV patients with cryptococcosis now outnumber PLWH patients with cryptococcosis in many institutions in the United States (36–56% vs 35–40%, respectively) [17,20,36,37]. The HIV-negative group of patients with cryptococcosis is highly heterogeneous, including both immunocompetent (13–39%) and immunocompromised persons (61–87%). [17,19,30,32,38] Thus, it is vital that physicians are familiar with factors associated with increased risk of delays in diagnosis in these individuals.

Several underlying comorbidities, including congestive heart failure, chronic liver disease, cancer, chronic pulmonary disease, and lymphoma, were associated with increased risk of missed diagnosis of cryptococcosis in both PLWH and HIV-negative persons. This is likely due to one of two possible reasons. First, many of these are risk factors for development of cryptococcosis [35], however, persons with these conditions do not fit into the classical high risk populations for cryptococcosis. Second, the early symptoms of cryptococcosis are nondescript and pleomorphic and can easily be attributed to another disease or condition. For instance, a new diagnosis of pulmonary nodule may not be malignancy but could be the evolution of cryptococcosis [39]. Episodes of hepatic encephalopathy or culture-negative spontaneous bacterial peritonitis presenting as non-differentiating sepsis in cirrhotic patients could be due to cryptococcosis [12,19]. It is possible that patients are misdiagnosed or miscoded with another condition with similar symptoms, and for this reason, clinicians should keep a high index of suspicion for cryptococcosis.

Possible reasons for this delay from presentation to diagnosis could be atypical presentations of cryptococcosis, especially in HIV-negative patients [19], and lack of experience identifying these complex phenotypes. The clinical presentations of cryptococcosis differ based on immune status [19]. For example, approximately 30% of HIV-negative patients have meningitis, compared to 70% in PLWH. Fever is present in only about 50% of HIV-negative patients, compared to 70% of PLWH [6,19]. A recent study of cryptococcal meningitis reported significant delays in diagnosis driven by knowledge deficits, leading to a failure to consider cryptococcosis in the differential diagnoses even in common syndromes such as meningitis [23,25]. Though states with high rates of cryptococcosis hospitalization independent of HIV status, such as Florida, Maryland and New York (>4 hospitalizations per million population) [33], were included in the analysis, no association between state and missed diagnosis was found.

In our study, lumbar puncture was coded in 14·6% of patients with a potentially missed diagnosis (Supplementary Table 2), suggesting that suspicion for meningitis was raised, but cryptococcosis was not identified. Incorporating CrAg screening of cerebrospinal fluid or serum as a part of routine practice could help improve diagnosis rates of cryptococcosis [10]. CrAg testing is highly sensitive (98–100%) and specific (99–100%) for diagnosis of cryptococcal meningitis in both PLWH and HIV-negative patients [3,[39], [40], [41]]. As such, the test should be used with serum for patients who present with other syndromes that overlap with cryptococcosis. The test yields results within 15 min, and with an estimated total cost of $5·43 per test [8], it is highly cost- and time-effective [42], and simple to use [41]. Thus, there are limited barriers for implementation of routine CrAg testing in patients with syndromes compatible with cryptococcosis, for example, all adults with suspected meningitis and pneumonia that do not respond to antibiotics. While these clinical presentations have a wide differential diagnoses, dissemination is often under evaluated in pulmonary cryptococcosis [23] with lower mortality seen in pulmonary cryptococcosis has than cryptococcal meningitis [21]. The CrAg screen-and-treat strategy is a highly effective intervention and key to preventing progression to more severe disease and lowering cryptococcosis-related mortality [8,9].

Overall, our results are generalizable across a variety of settings. The data used for our analysis was collected from 8 different states, comprising roughly 30% of the country, and representing a wide variety of geographic regions spanning the range of rate of diagnosis, covering low, middle, and high prevalence areas. Due to the pervasive nature of cryptococcus worldwide, the results of our study are important for global practice. Lastly, recognition of cryptococcosis, especially in low prevalence areas, is important for general practice, considering that a patients first encounter is likely not with an infectious disease specialist.

Our study is limited by its retrospective design and the nature of the administrative database. Additionally, the SID does not include laboratory results or consulting physician specialty (i.e. infectious diseases), limiting our analysis into effects of consultation by different specialties and differences in laboratory characteristics (ie, CrAg) between groups. Furthermore, it is likely that our study underestimated the proportion of patients with cryptococcosis with missed or delayed diagnosis as we did not have access to outpatient records, where patients may have initially sought care. Additionally, because the database only captures in-hospital mortality, patients who died without receiving a diagnosis or received only postmortem diagnosis, were not included in our analysis, further underestimating the magnitude of missed opportunities. We also did not have access to out-of-hospital mortality data, and therefore the mortality accounted for in our study is likely an underestimate of the true 90-day death rate. Combined, these limitations are likely to underestimate the magnitude of the effect observed.

Missed opportunities to diagnose cryptococcosis in the hospital setting are common, particularly among HIV-negative patients. Potentially missed diagnoses were associated with increased risk of 90-day mortality in HIV-negative patients. This emphasizes the need for a higher index of suspicion for cryptococcosis, as well as highlights the importance of early CrAg testing.

## Author contributions

All authors had full access to all of the data in the study and take responsibility for the accuracy of the data and the integrity of the data analysis. ASS, MAO, IAG, WGP, and AS were responsible for study concept and design. All authors were responsible for data acquisition, definition of variables, and interpretation of results. ASS, MAO, KBN, LL, and AS were responsible for drafting of the manuscript. MK, MAO, and KBN were responsible for statistical analysis. All authors were responsible for critical revision of the manuscript for important intellectual content. MAO, and AS obtained funding.

## Declaration of Competing Interest

AS reports grants and personal fees from Astellas Global Development Pharma and Mayne Pharma; grants from Scynexis, Cidara, MiraVista, and IMMY; and personal fees from Viamet, outside the submitted work. WGP reports grants and personal fees from Merck and Co, and Gilead Sciences. MK and KBN report grants from NIH; and grants from AHRQ. MAO reports grants from NIH/NCATS; grants from AHRQ, Pfizer; and grants and personal fees from Merck and Sanofi Pasteur, outside the submitted work. All other authors declare no competing interests.

## Appendix Supplementary materials

Image, application 1

Image, application 2
