# Supplementary material for: Potential missed opportunities for diagnosis of cryptococcosis and the association with mortality: A cohort study
Source: eClinicalMedicine. 2020 Oct 7;27:100563. doi: 10.1016/j.eclinm.2020.100563 (PMC7648127; doi:10.1016/j.eclinm.2020.100563)
Supplement: Supplementary file 1 [file mmc1.docx]

**Supplementary Appendix**

**Supplementary Table 1:** ICD-9-CM Procedure and Diagnosis Codes Used to Define Central Nervous System Conditions, Pulmonary Conditions, and Non-Specific Signs of Infection Suggestive of Cryptococcosis

**Supplementary Table 2.** Hierarchy-Based Definition of a Potential Missed Opportunity to Diagnose Cryptococcosis at the Individual Level (N=1,470 People)

**Supplementary Table 3:** ICD-9-CM Diagnosis Codes Used to Create Additional Comorbidities Associated with Cryptococcosis

**Supplementary Table 4:** Univariate Analysis of Risk for Missed Diagnosis within 90 Days Prior to the Cryptococcosis Admission by HIV Status

**Supplementary Table 5:** Univariate Analysis of Risk for Mortality within 90 Days After the Cryptococcosis Admission by HIV Status.

| **Supplementary Table 1. ICD-9-CM Procedure and Diagnosis Codes Used to Define Central Nervous System Conditions, Pulmonary Conditions, and Non-Specific Signs of Infection Suggestive of Cryptococcosis** | | |
| --- | --- | --- |
| **Category** | **ICD-9-CM Diagnosis Code** | **ICD-9-CM Procedure Code** |
| **Central Nervous System** | | |
| **Lumbar Puncture** |  | 03.31 |
| **Increased Intracranial Pressure** | 331.3, 331.4, 331.5 | 88.91 |
| **Brain Infection** | 047.9, 320.7, 320.9, 321.4, 322.2, 322.9, 323.82, 326, V12.42 | 88.93 |
| **Encephalopathy** | 070.20, 070.21, 070.22, 070.44, 070.71, 290.3, 293.1, 298.9, 323.8, 323.81, 3348.30, 348.31, 348.39, 349.82, 572.2, 780.01, 780.09, 780.1, 780.97 | 02.22, 02.32, 02.34, 02.42, 02.43 |
| **Headache** | 307.81, 339.3, 346.10, 346.70, 346.90, 346.91, 784.0 | 01.12, 03.90 |
| **Focal Neurological Deficit** | 368.8, 389.12, 389.9, 438.84, 780.2, 780.39, 780.4, 781.0, 781.2, 781.3, 784.3, 7845 | 01.10, 01.13, 01.14, 01.28, 01.31 01.39, 01.59, 02.2, 03.09, 03.92 |
| **Stroke** | 435.8, 435.9, V12.54 | 01.51 |
| **Other** | 348.5, 348.89, 348.9, 348.1 |  |
| **Pulmonary** | | |
| **Pneumonia Not Otherwise Specified** | 483.8, 484.7, 485, 486, 490, 493.22, 494.0, 496, 516.36, 516.8-518.84, 714.81, 786.05, 786.09, 786.2, 793.1, 793.11, V12.61 | 32.20, 32.29, 32.30, 32.49, 33.21-33.24, 33.26-33.28 |
| **Pleural Disease** | 511.9 | 34.04, 34.06, 34.91, 88.73 |
| **Mediastinal Diagnosis** |  | 34.22 |
| **Other** | 518.89, 786.52 |  |
| **Non-Specific Signs of Infection** | | |
| **Signs and Symptoms of Infection** | 780.6, 780.60, 780.61, 780.79, 785.0, 785.6 |  |
| **Infection Not Otherwise Specified** | 079.99, 117.9, 567.23, 785.52, 725, 995.90-995.92 |  |

| **Supplementary Table 2: Hierarchy-Based Definition of a Potential Missed Opportunity to Diagnose Cryptococcosis * at the Individual Level (N=1,470 People)** | | |
| --- | --- | --- |
| **Central Nervous System Conditions** | **Frequency** | **Percent** |
| Lumbar Puncture | 214 | 14.6 |
| Increased Intracranial Pressure | 28 | 1.9 |
| Brain Infection | 24 | 1.6 |
| Encephalopathy | 235 | 16.0 |
| Headache | 93 | 6.3 |
| Focal Neurological Deficit | 140 | 9.5 |
| Stroke | 33 | 42.2 |
| Other | 11 | 0.7 |
| **Total** | 778 | 52.9 |
| **Pulmonary Conditions** | **Frequency** | **Percent** |
| Pneumonia Not Otherwise Specified | 490 | 33.3 |
| Mediastinal and Pleural Disease | 65 | 4.4 |
| Other | 20 | 1.4 |
| **Total** | 575 | 39.1 |
| **Non-Specific Signs of Infection** | **Frequency** | **Percent** |
| Presumed Infection | 66 | 4.5 |
| Infection Not Otherwise Specified | 51 | 3.5 |
| **Total** | 117 | 8.0 |
| ^*^A missed opportunity to diagnose cryptococcosis was defined hierarchically based on three main conditions (central nervous system conditions, pulmonary conditions, and non-specific signs of infection). Within each condition, procedure and diagnosis codes were ordered hierarchically into subcategories based on the level of suspicion of cryptococcosis. | | |

| **Supplementary Table 3: ICD-9-CM Diagnosis Codes Used to Create Additional Comorbidities Associated with** Cryptococcosis | |
| --- | --- |
| Leukemias | 202.40-202.48, 203.10-203.12, 204.00-204.02, 204.10-204.12, 204.-204.22, 204.80-204.82, 204.90-204.92, 205.00-205.02, 205.10-205.12, 205.20- 205.22, 205.3 |
| Cellular Immunodeficiency | 279.8, 279.9, 279.01,279.49 |
| History of Solid Organ Transplant | V42.0, V42.1, V42.6, V42.7, V42.81-V42.84, 556.9, 505.9, 375.1, 335.0-335.2, 336, 528.0, 469.7 |
| Other Neurological Disorders * | 330.0-331.9, 334.0-335.9, 341.1-341.9, 345.00-345.11, 345.2, 345.3, 345.40-345.91, 768.70-768.73, 780.31-780.33, 780.39, 780.97, 784.3 |
| Autoimmune Disorders * | 696.0, 696.1, 696.8, 446.0, 446.1, 446.4, 446.5, 446.7, 446.20, 446.29, 437.4, 447.6, 710.2, 695.4, 710.0, 135, 255.0 |
| ^*^ Two Elixhauser variables had to be modified to avoid collinearity with the additional variables. Codes for altered mental status, cerebral degeneration, convulsions, aphasia, and cerebral degeneration were removed from “neurological disorders” variable and included in the other neurological disorder variable. The code for systemic lupus erythematous was removed from the “rheumatoid arthritis” variable and included in the new autoimmune disorder variable. | |

| **Supplementary Table 4: Univariate Analysis of Risk for Missed Diagnosis within 90 Days Prior to the Cryptococcosis Admission by HIV Status** | | | | | | |
| --- | --- | --- | --- | --- | --- | --- |
|  | **PLHIV** | | | **HIV NEGATIVE** | | |
|  | **Missed N=493** | **Not Missed N=1,952** | **P** | **Missed N=977** | **Not Missed N=1,932** | **P** |
|  | **N (%)** | **N (%)** |  | **N (%)** | **N (%)** |  |
| **Female** | 105 (21.3) | 326 (16.7) | 0.017 | 408 (41.8) | 696 (36.0) | 0.003 |
| **Race/Ethnicity** |  |  | 0.069 |  |  | 0.652 |
| **White** | 122 (24.8) | 507 (26.0) |  | 524 (53.6) | 1003 (51.9) |  |
| **Black** | 244 (49.5) | 832 (42.6) |  | 134 (13.7) | 296 (15.3) |  |
| **Hispanic** | 107 (21.7) | 511 (26.2) |  | 189 (19.3) | 354 (18.3) |  |
| **Other** | 20 (4.1) | 102 (5.2) |  | 130 (13.3) | 280 (14.4) |  |
| **Year** |  |  | 0.001 |  |  | 0.102 |
| **2005** | 50 (10.1) | 282 (14.5) |  | 66 (6.8) | 167 (8.6) |  |
| **2006** | 58 (11.8) | 293 (15.0) |  | 105 (10.8) | 231 (12.0) |  |
| **2007** | 68 (13.79) | 265 (13.6) |  | 114 (11.7) | 196 (10.1) |  |
| **2008** | 65 (13.2) | 227 (11.6) |  | 102 (10.4) | 201 (10.4) |  |
| **2009** | 58 (11.8) | 228 (11.7) |  | 116 (11.9) | 186 (9.6) |  |
| **2010** | 43 (8.7) | 185 (9.5) |  | 117 (12.0) | 182 (9.4) |  |
| **2011** | 37 (7.5) | 169 (8.7) |  | 98 (10.0) | 200 (10.4) |  |
| **2012** | 15 (3.0) | 60 (3.1) |  | 65 (6.5) | 123 (6.4) |  |
| **2013** | 27 (5.5) | 90 (4.6) |  | 74 (7.6) | 161 (8.3) |  |
| **2014** | 44 (8.9) | 103 (5.3) |  | 68 (7.0) | 166 (8.6) |  |
| **2015** | 28 (5.7) | 50 (2.6) |  | 52 (5.3) | 119 (6.2) |  |
| **Metastatic Cancer** | ·· * | ·· * | <0.001 | 67 (6.9) | 65 (3.4) | <0.001 |
| **Medicaid/Uninsured vs. Other** ^†^ | 295 (59.8) | 1025 (52.5) | 0.003 | 194 (19.9) | 401 (20.8) | 0.570 |
| **Other Neurological Disorders** ^‡^ | 46 (9.3) | 78 (4.0) | <0.001 | 93 (9.5) | 94 (4.9) | <0.001 |
| **Autoimmune Disorders ^‖^** | ·· * | 40 (2.1) | 0.801 | 130 (13.3) | 174 (9.0) | <0.001 |
| **Valvular Disorders** | 19 (3.9) | 35 (1.8) | 0.005 | 92 (9.4) | 138 (7.1) | 0.032 |
| **Congestive Heart Failure** | 42 (8.5) | 54 (2.8) | <0.001 | 261 (26.7) | 246 (12.7) | <0.001 |
| **Chronic Liver Disease** | 87 (17.7) | 168 (8.6) | <0.001 | 211 (21.6) | 236 (12.2) | <0.001 |
| **Hypertension** | 187 (37.9) | 483 (24.7) | <0.001 | 693 (70.9) | 1,053 (54.5) | <0.001 |
| **Pulmonary Circulation Disorders** | 27 (5.5) | 39 (2.0) | <0.001 | 121 (12.4) | 103 (5.3) | <0.001 |
| **Peripheral Vascular Disease** | ·· * | 16 (0.8) | 0.007 | 104 (10.4) | 92 (4.7) | <0.001 |
| **Chronic Pulmonary Disease** | 112 (22.7) | 174 (8.9) | <0.001 | 316 (32.3) | 333 (17.2) | <0.001 |
| **Diabetes Mellitus** | 68 (13.8) | 160 (8.2) | 0.001 | 438 (44.8) | 616 (31.9) | <0.001 |
| **Hypothyroidism** | 16 (3.3) | 27 (1.4) | 0.005 | 154 (15.8) | 184 (9.5) | <0.001 |
| **Lymphoma** | 21 (4.3) | 32 (1.4) | <0.001 | 110 (11.3) | 119 (6.2) | <0.001 |
| **Solid Tumor without Metastasis** | 21 (4.3) | 83 (4.3) | 0.994 | 49 (5.0) | 81 (4.2) | 0.310 |
| **Rheumatoid Arthritis/ Collagen Vascular Diseases‖** | ·· * | ·· * | 0.268 | 75 (7.7) | 97 (5.0) | 0.004 |
| **Weight Loss** | 199 (40.4) | 513 (26.3) | <0.001 | 329 (33.7) | 313 (16.2) | <0.001 |
| **Obesity** | 20 (4.06) | 36 (1.84) | 0.003 | 137 (14.0) | 152 (7.9) | <0.001 |
| **Deficiency Anemia** | 308 (62.5) | 844 (43.2) | <0.001 | 567 (58.0) | 649 (33.6) | <0.001 |
| **Drug Abuse** | 164 (33.3) | 336 (17.2) | <0.001 | 73 (7.5) | 96 (5.0) | 0.006 |
| **Alcohol Abuse** | 79 (16.0) | 146 (7.5) | <0.001 | 87 (8.9) | 87 (4.5) | <0.001 |
| **Renal Failure** | 70 (14.2) | 147 (7.5) | <0.001 | 358 (36.6) | 468 (24.2) | <0.001 |
| **Leukemia** | ·· * | ·· * | 0.615 | 79 (8.1) | 103 (5.3) | 0.004 |
| **Immunodeficiencies** | ·· * | ·· * | 0.036 | 34 (3.5) | 43 (2.2) | 0.047 |
| **History of Solid Organ Transplant** | ·· * | ·· * | 0.047 | 128 (13.1) | 204 (10.6) | 0.042 |
| * Healthcare Cost and Utilization Project privacy protection requirements prohibit reporting cell sizes less than or equal to 10.  ^†^ Other type of Insurance includes: Private, Medicare, Self-pay.  ^‡^ Other neurological disorders include: altered mental status, aphasia, convulsions, cerebral degeneration. ^§^ Autoimmune disorders include: psoriatic arthritis, psoriasis, reactive arthritis, Kawasaki, Takayasu, granulomatosis w/polyangiitis, eosinophilic (Wegener) giant cell arteritis, hypersensitivity angitis, Cerebral arthritis, arteritis not otherwise specified, Sjogren, Inflammatory bowel disease, lupus, sarcoid, Cushing disease.  **^‖^** Modified from Elixhauser - domain does not include Systemic Lupus. | | | | | | |

| **Supplementary Table 5: Univariate Analysis of Risk for Mortality within 90 Days After the Cryptococcosis Admission by HIV Status** | | | | | | |
| --- | --- | --- | --- | --- | --- | --- |
|  | **PLHIV** | | | **HIV NEGATIVE** | | |
|  | **Mortality N=348** | **No Mortality  N=2,097** | **P** | **Mortality N=534** | **No Mortality  N=2,375** | **P** |
|  | **N (%)** | **N (%)** |  | **N (%)** | **N (%)** |  |
| **Female** | 63 (18.1) | 368 (17.6) | 0.802 | 201 (37.6) | 903 (38.0) | 0.870 |
| **Race/Ethnicity** |  |  | 0.322 |  |  | 0.358 |
| **White** | 84 (24.1) | 545 (26.0) |  | 269 (50.4) | 1,258 (53.0) |  |
| **Black** | 162 (46.6) | 914 (43.6) |  | 84 (15.7) | 346 (14.6) |  |
| **Hispanic** | 85 (24.4) | 533 (25.4) |  | 109 (20.4) | 434 (18.3) |  |
| **AI/PI** | 8 (2.3) | 26 (1.2) |  | 36 (6.7) | 136 (5.7) |  |
| **Other** | 9 (2.6) | 79 (3.8) |  | 36 (6.7) | 201 (8.5) |  |
| **Year** |  |  | 0.701 |  |  | 0.675 |
| **2005** | 42 (12.1) | 290 (13.8) |  | 43 (8.1) | 190 (8.0) |  |
| **2006** | 61 (17.5) | 290 (13.8) |  | 63 (11.8) | 273 (11.5) |  |
| **2007** | 43 (12.4) | 290 (13.8) |  | 71 (13.3) | 239 (10.1) |  |
| **2008** | 43 (12.4) | 249 (11.9) |  | 55 (10.3) | 248 (10.4) |  |
| **2009** | 39 (11.2) | 247 (11.8) |  | 58 (10.9) | 244 (10.3) |  |
| **2010** | 30 (8.6) | 198 (9.4) |  | 53 (9.9) | 246 (10.4) |  |
| **2011** | 32 (9.2) | 174 (8.3) |  | 50 (9.4) | 248 (10.4) |  |
| **2012** | 8 (2.3) | 67 (3.2) |  | 30 (5.6) | 158 (6.7) |  |
| **2013** | 15 (4.3) | 102 (4.9) |  | 38 (7.1) | 197 (8.3) |  |
| **2014** | 20 (5.8) | 127 (6.1) |  | 46 (8.6) | 188 (7.9) |  |
| **2015** | 15 (4.3) | 63 (3.0) |  | 27 (5.1) | 144 (6.1) |  |
| **Missed vs. Not Missed** | 83 (23.9) | 410 (19.6) | 0.064 | 236 (44.2) | 741 (31.2) | <0.001 |
| **Congestive Heart Failure** | 34 (9.8) | 62 (3.0) | <0.001 | 141 (26.4) | 366 (15.4) | <0.001 |
| **Chronic Liver Disease** | 52 (14.9) | 203 (9.7) | 0.003 | 141 (26.4) | 306 (12.9) | <0.001 |
| **Hypertension** | 129 (37.1) | 541 (25.8) | <0.001 | 341 (63.9) | 1,405 (59.2) | 0.045 |
| **Metastatic Cancer** | ·· * | ·· * | 0.034 | 33 (6.2) | 99 (4.2) | 0.044 |
| **Medicaid/Uninsured vs. Other** ^†^ | 194 (55.8) | 1,126 (53.7) | 0.477 | 106 (19.9) | 489 (20.6) | 0.702 |
| **Other Neurological Disorders** ^‡^ | 33 (9.5) | 91 (4.3) | <0.001 | 37 (6.9) | 150 (6.3) | 0.602 |
| **Autoimmune Disorders** ^§^ | ·· * | 43 (2.1) | 0.764 | 44 (8.2) | 260 (11.0) | 0.065 |
| **Valvular Disorders** | ·· * | 46 (2.2) | 0.902 | 59 (11.1) | 171 (7.2) | 0.003 |
| **Pulmonary Circulation Disorders** | 16 (4.6) | 50 (2.4) | 0.018 | 57 (10.7) | 167 (7.0) | 0.004 |
| **Peripheral Vascular Disease** | 13 (3.7) | 14 (0.7) | <0.001 | 42 (7.9) | 153 (6.4) | 0.235 |
| **Chronic Pulmonary Disease** | 44 (12.6) | 242 (11.5) | 0.553 | 130 (24.3) | 519 (21.9) | 0.211 |
| **Diabetes Mellitus** | 46 (13.2) | 182 (8.7) | 0.007 | 210 (39.3) | 844 (35.5) | 0.1 |
| **Hypothyroidism** | ·· * | 36 (1.7) | 0.699 | 71 (13.3) | 267 (11.2) | 0.181 |
| **Lymphoma** | ·· * | 43 (2.1) | 0.329 | 51 (9.6) | 178 (7.5) | 0.111 |
| **Solid Tumor without Metastasis** | 14 (4.0) | 90 (4.3) | 0.818 | 35 (6.6) | 95 (4.0) | 0.01 |
| **Rheumatoid Arthritis/Collagen Vascular Diseases ^‖^** | ·· * | ·· * | 0.099 | 31 (5.8) | 141 (5.9) | 0.907 |
| **Obesity** | ·· * | 45 (2.2) | 0.241 | 53 (9.9) | 236 (9.9) | 0.994 |
| **Deficiency Anemia** | 177 (50.9) | 975 (46.5) | 0.131 | 256 (47.9) | 960 (40.4) | 0.002 |
| **Weight Loss** | 143 (41.1) | 569 (27.1) | <0.001 | 178 (33.3) | 464 (19.5) | <0.001 |
| **Drug Abuse** | 74 (21.3) | 426 (20.3) | 0.684 | 26 (4.9) | 143 (6.0) | 0.304 |
| **Alcohol Abuse** | 34 (9.8) | 191 (9.1) | 0.692 | 54 (10.1) | 120 (5.0) | <0.001 |
| **Renal Failure** | 43 (12.4) | 172 (8.2) | 0.011 | 173 (32.4) | 653 (27.5) | 0.023 |
| **Psychosis** | 27 (7.8) | 169 (8.1) | 0.848 | 31 (5.8) | 129 (5.4) | 0.732 |
| **Depression** | 34 (9.8) | 267 (12.7) | 0.119 | 54 (10.1) | 281 (11.8) | 0.261 |
| **Leukemia** | ·· * | ·· * | 0.014 | 41 (7.7) | 141 (5.9) | 0.133 |
| **Immunodeficiencies** | ·· * | ·· * | 0.625 | ·· * | 66 (2.8) | 0.35 |
| **History of Solid Organ Transplant** | ·· * | ·· * | 0.014 | 40 (7.5) | 292 (12.3) | 0.002 |
| * Healthcare Cost and Utilization Project privacy protection requirements prohibit reporting cell sizes less than or equal to 10.  ^†^ Other type of Insurance includes: Private, Medicare, Self-pay.  ^‡^ Other neurological disorders include: altered mental status, aphasia, convulsions, cerebral degeneration. ^§^ Autoimmune disorders include: psoriatic arthritis, psoriasis, reactive arthritis, Kawasaki, Takayasu, granulomatosis w/polyangiitis, eosinophilic (Wegener) giant cell arteritis, hypersensitivity angitis, Cerebral arthritis, arteritis not otherwise specified, Sjogren, Inflammatory bowel disease, lupus, sarcoid, Cushing disease.  **^‖^** Modified from Elixhauser - domain does not include Systemic Lupus. | | | | | | |
